# Can Immune Tolerance Be Re-established in Neuromyelitis Optica?

**DOI:** 10.3389/fneur.2021.783304

**Published:** 2021-12-20

**Authors:** Eileah Loda, Gabriel Arellano, Gina Perez-Giraldo, Stephen D. Miller, Roumen Balabanov

**Affiliations:** ^1^Department of Microbiology-Immunology, Feinberg School of Medicine, Northwestern University, Chicago, IL, United States; ^2^Department of Neurology, Northwestern University, Chicago, IL, United States

**Keywords:** neuromyelitis optica (NMO), immune tolerance, PLG nanoparticles, NMOSD, autoimmune disease, tolerogenic immune-modifying nanoparticles, disease-modifying therapies

## Abstract

Neuromyelitis optica (NMO) is a chronic inflammatory disease of the central nervous system that primarily affects the optic nerves and spinal cord of patients, and in some instances their brainstem, diencephalon or cerebrum as spectrum disorders (NMOSD). Clinical and basic science knowledge of NMO has dramatically increased over the last two decades and it has changed the perception of the disease as being inevitably disabling or fatal. Nonetheless, there is still no cure for NMO and all the disease-modifying therapies (DMTs) are only partially effective. Furthermore, DMTs are not disease- or antigen-specific and alter all immune responses including those protective against infections and cancer and are often associated with significant adverse reactions. In this review, we discuss the pathogenic mechanisms of NMO as they pertain to its DMTs and immune tolerance. We also examine novel research therapeutic strategies focused on induction of antigen-specific immune tolerance by administrating tolerogenic immune-modifying nanoparticles (TIMP). Development and implementation of immune tolerance-based therapies in NMO is likely to be an important step toward improving the treatment outcomes of the disease. The antigen-specificity of these therapies will likely ameliorate the disease safely and effectively, and will also eliminate the clinical challenges associated with chronic immunosuppressive therapies.

## Introduction

Neuromyelitis optica (NMO) is a rare autoimmune disorder mediated by self-reactive T and B cells, and an antibody against the aquaporin 4 (AQP4) channel protein of astrocytes (1). Clinically, this is a chronic inflammatory disorder of the central nervous system (CNS) that affects primarily the optic nerves and spinal cord of patients, causing optic neuritis and transverse myelitis (2). Despite the overlapping symptomology with multiple sclerosis (MS), NMO is an independent neurological entity with distinct clinicopathological characteristics (3). NMO spectrum disorders (NMOSD) are a broad clinical category that encompasses the classic optico-spinal syndrome and its partial presentation, together with some rare neurological syndromes (brain stem, diencephalon, area postrema, and cerebrum) involving the anti-AQP4 antibody, as well as their seronegative presentations (4, 5). Typically, NMOSD are idiopathic conditions, but they can also occur in the context of connective tissue, paraneoplastic and infectious diseases (6, 7). The seronegative category of NMOSD often differs clinically from its seropositive counterpart and it is commonly associated with anti-myelin oligodendrocyte glycoprotein (MOG) antibody (8, 9). This category would likely require future redefinition because of its considerable phenotypic overlap with MOG antibody disorders (MOGAD), which are demyelinating and not astrocytopathic entities (9).

Research over the last two decades has led to new understanding of NMO pathogenesis, which naturally evolved into providing a rationale for immunotherapy of the disease. It became certain that therapeutic targeting of critical inflammatory mechanisms of the disease can change its natural course and improve patients' prognosis (10, 11). Importantly, advancement of therapeutic modalities changed the perception of the disease as being inevitably disabling or fatal. It also became apparent that the available disease-modifying therapies (DMTs) are often associated with serious adverse effects and might have prohibitive financial cost (11). Furthermore, the unpredictable activity of NMO, the partial efficacy of DMTs and their adverse effect profile create clinical challenges that cannot be fully addressed by the currently employed immunosuppressive or immunomodulatory strategies. One can hypothesize that a different approach, one distinguishing autoimmunity from protective immunity at the antigen level is likely to be most effective.

Immune tolerance is classically defined as a state of immune unresponsiveness to otherwise immunogenic molecules, cells or tissues (12). Immune tolerization is a therapeutic strategy focused on deliberate manipulation of the immune system and non-pathogenic introduction of the autoantigen, both aiming to prevent further self-reactive responses and treat autoimmunity (13). Its therapeutic significance to NMO has been previously recognized because the autoantigen AQP4 is a well-defined protein and the self-reactive response can be quantitatively measured (14–16). Another attractive point is that immune tolerization can be used either alone as a primary treatment modality, but also in conjunction with other immunotherapies when clinically indicated. Theoretically, AQP4 and, perhaps, MOG are reasonably good candidates for antigen-specific tolerization, providing that the necessary biotechnology is available.

In this review, we discuss the pathogenic mechanisms of NMO as they pertain to its DMTs and immune tolerance. We also examine a novel therapeutic strategy based on tolerogenic immune-modifying nanoparticles (TIMP) and its potential clinical significance (17). Research development and clinical implementation of such a strategy is likely to be an important step toward improving the treatment outcomes of NMO and resolve of the clinical challenges associated with chronic exposure to immunosuppression, safety and tolerability.

## Immunopathogenesis of NMO

NMO is a rare sporadic disease of the CNS with an overall incidence of about 1–4:100,000 population depending on the demographics of the studies (18, 19). The disease affects predominantly middle aged non-Caucasian (African-American, African, and Asian) females (19). In corroboration, the disease is most common in Africa and Asia, and in countries with a tropical climate (18, 19). However, other demographic groups such as Caucasian males and females, elderly or children can also be affected (18–20). There are reports of familial occurrence of the disease indicating the existence of hereditary risk factors (21). Genetic risk appears to be associated with certain HLA alleles including DQA1, DQB1, and DRB1, but not with DRB1^*^1501, the allele that is most often linked to multiple sclerosis. Whole-genome sequence analysis has identified DNA variations located in the major histocompatibility complex (MHC) region and a reduced copy number of the complement component 4A gene (22–24). Interestingly, the same analysis also provides evidence that NMO shares more genome-wide marker similarities with systemic lupus erythematosus than with MS (22–24).

High incidence of NMO in the tropics has led to speculation for an infectious cause of the disease (25). There are a number of reports indicating a prodromal association and co-occurrence of various infections with NMO and, notably, a positive correlation of some of them with the anti-AQP4 seropositivity (2, 26–30). Others have demonstrated the existence of structural homology and cross-immunoreactivity between human AQP4 and certain bacterial proteins (31, 32). In addition, Th17 cells (T helper cells producing interleukin 17) typically mediating host defense against microbial pathogens, are also critically involved in the pathogenesis of NMO (32–35). Hypothetically, a microbial infection and the resultant inflammatory reaction may compromise the processes of immune tolerance and trigger a self-reactive response. The autoimmune response is likely facilitated by the disease-associated HLA alleles, which might preferentially bias the antigen presentation toward homologous and cross-immunoreactive epitopes.

**Figure 1:**
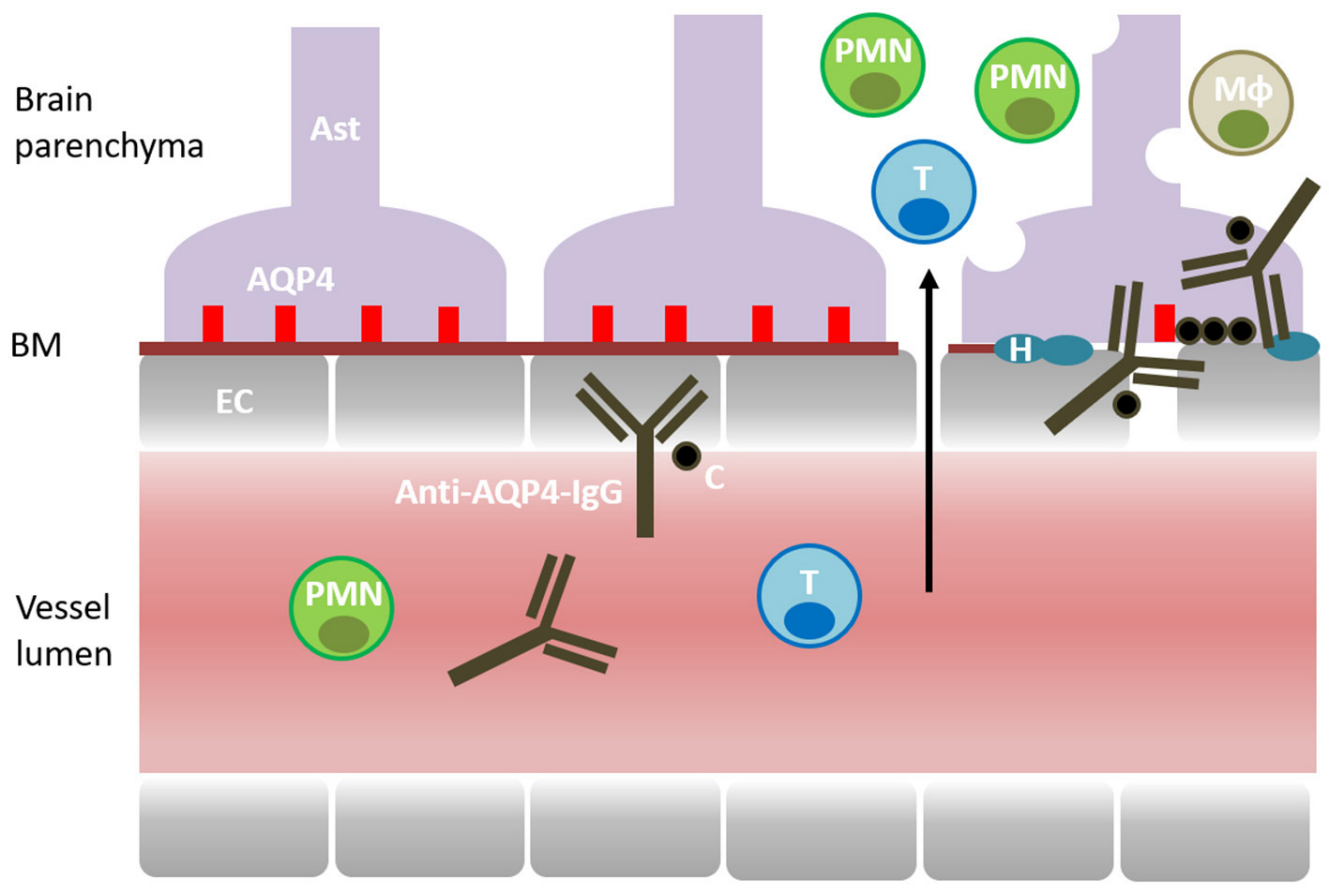
Pathogenesis of NMO. Aquaporin 4 (AQP4) is expressed by the CNS astrocytes (AST) and localized at their perivascular processes, immediately beyond the basement membrane (BM) and the endothelial cells (EC). The disease is mediated by a self-reactive antibody targeting AQP4 (Anti-AQP4-IgG). Complement (C) is recruited and activated by the autoantibody. Cellular inflammation involves T cells (T), macrophages (Mφ), polymorphonuclear cells (PMN) (neutrophils and eosinophils). Inflammatory reaction causes astrocyte cell injury, downregulation of AQP4 and accumulation of hyaline (H) in the blood vessel walls. Ultimately, inflammation leads to vasogenic edema, secondary demyelination, tissue damage and loss of function.

Anti-AQP4 antibody is the principal effector molecule in NMO pathogenesis (36, 37). The autoantibody is a self-reactive IgG1 targeting human AQP4 in the CNS (36) (Figure 1). AQP4 is a 35kDa water channel protein that is expressed by CNS astrocytes and it is highly clustered at astrocytic processes surrounding the small blood vessels and the brain ependyma (10). Localization of AQP4 immediately beyond the basement membrane of blood vessels and ependyma makes it an accessible target for the circulating autoantibody. The autoantibody has the capacity to bind to AQP4 on the astrocytic foot processes, to activate complement, and to recruit mononuclear and polymorphonuclear cells to the site of initial antigen recognition (38). Independently, autoantibody-AQP4 interaction has the capacity to induce astrocyte activation, to upregulate the expression of interleukin 6 (IL-6) and other pro-inflammatory molecules, and to independently accelerate the inflammatory reaction (39). In addition to antigen binding and complement activation, the anti-AQP4 antibody can engage the Fc receptors of the NK (natural killer) cells and monocytes, and trigger antibody-dependent cellular cytotoxicity (ADCC) (40). The autoimmune targeting of AQP4 ultimately leads to astrocyte cell injury and downregulation of a number of structural and functional proteins involved in the maintenance of local homeostasis including AQP4, glial fibrillary acid protein (GFAP) and glutamate transporter excitatory amino acid transporter 2 (EAAT2) (41–44). As the autoimmune reaction unfolds, it loses its initial specificity and the ensuing inflammation becomes dominated by vasogenic edema, oxidative stress, excitotoxicity, secondary demyelination and tissue necrosis.

Various studies also indicate that Th17 cells specific to AQP4 may also be involved in the disease pathogenesis (34, 35). Th17 cytokines are implicated in the breakdown of the blood-brain barrier, thereby, allowing the extravasation of the anti-AQP4 antibody and complement as well as the recruitment of mononuclear and polymorphonuclear cells to the lesion site. Clinical evidence also indicates that autoimmunity in NMO is dependent on the balance of Th1/Th17 immune responses and medications that promote a Th17 bias are likely to cause a disease relapse (45, 46). For instance, IFN-β that is commonly used in the treatment of multiple sclerosis based on its downregulatory effects on Th1 cells (T helper cells producing INF-γ) increases anti-AQP4 antibody titers and disease activity in NMO (45, 46). In contrast, therapeutic blockade of IL-6, a cytokine that potentiates differentiation of Th17 immune responses, is effective in suppressing disease activity in patients (47).

There is insufficient knowledge about the mechanisms underlying the unique involvement of optic nerves and spinal cord in NMO. Anatomically, the blood-brain barrier is incomplete in the pre-laminar portion of the optic nerves and the nerve roots of the spinal cord, as well as in the area postrema and diencephalon, which might allow unrestricted access of the autoantibody and related inflammatory molecules to the CNS. Another important factor appears to be the relative abundance of M23 isoform of AQP4 specifically in these structures (48). M23 isoform is critical for the formation of the AQP4 supramolecular complexes, which represents the principle conformational target of the anti-AQP4 antibody (49). Additional consideration is the marked downregulation of the complement activation regulatory molecules (CD46, CD55, and CD59) in the NMO lesions (50). These membrane-bound molecules do not co-localize topographically with AQP4 at the astrocytic processes, which creates a predisposition for a subthreshold complement activation by the autoantibody. This is in contrast to the peripheral organs, typically spared in the disease, where these molecules are co-expressed and co-localized with AQP4 (50, 51).

Anti-MOG antibody has been implicated in the pathogenesis of NMOSD where anti-AQP4 is not detected (seronegative NMOSD) (7, 9). The prevalence of anti-MOG antibody positivity in seronegative NMOSD is rather significant and can vary between 10 and 45%. The findings are likely reflective of a phenotypic overlap between seronegative NMOSD and MOGAD and the existence of shared pathogenic pathways and similar, but not identical targets. MOGAD as a diagnostic category that comprises of a number of demyelinating disorders including, isolated optic neuritis, transverse myelitis, acute disseminated encephalomyelitis and tumefactive MS, that might satisfy clinically the diagnostic criteria of NMOSD (7, 9). The differential diagnosis between NMOSD and MOGAD is challenging and largely depends on the detection of anti-AQP4 and anti-MOG antibodies, symptom monitoring and periodic revisiting of the clinical history and diagnosis or in some cases on lesion biopsy. Pathologically, MOGAD appear to be distinct from NMOSD in that they are primarily demyelinating and not astrocytopathic entities (52). Specifically, MOGAD are characterized by various levels of oligodendrocyte destruction, possibly loss of MOG expression, but also by preservation of astrocytic morphology and AQP4 expression (52, 53). Pathological findings also indicate prominent CD4+ T cell and macrophage involvement as well as with granulocyte infiltration and complement deposition (52, 53).

## Immunotherapy of NMO

**Figure 2:**
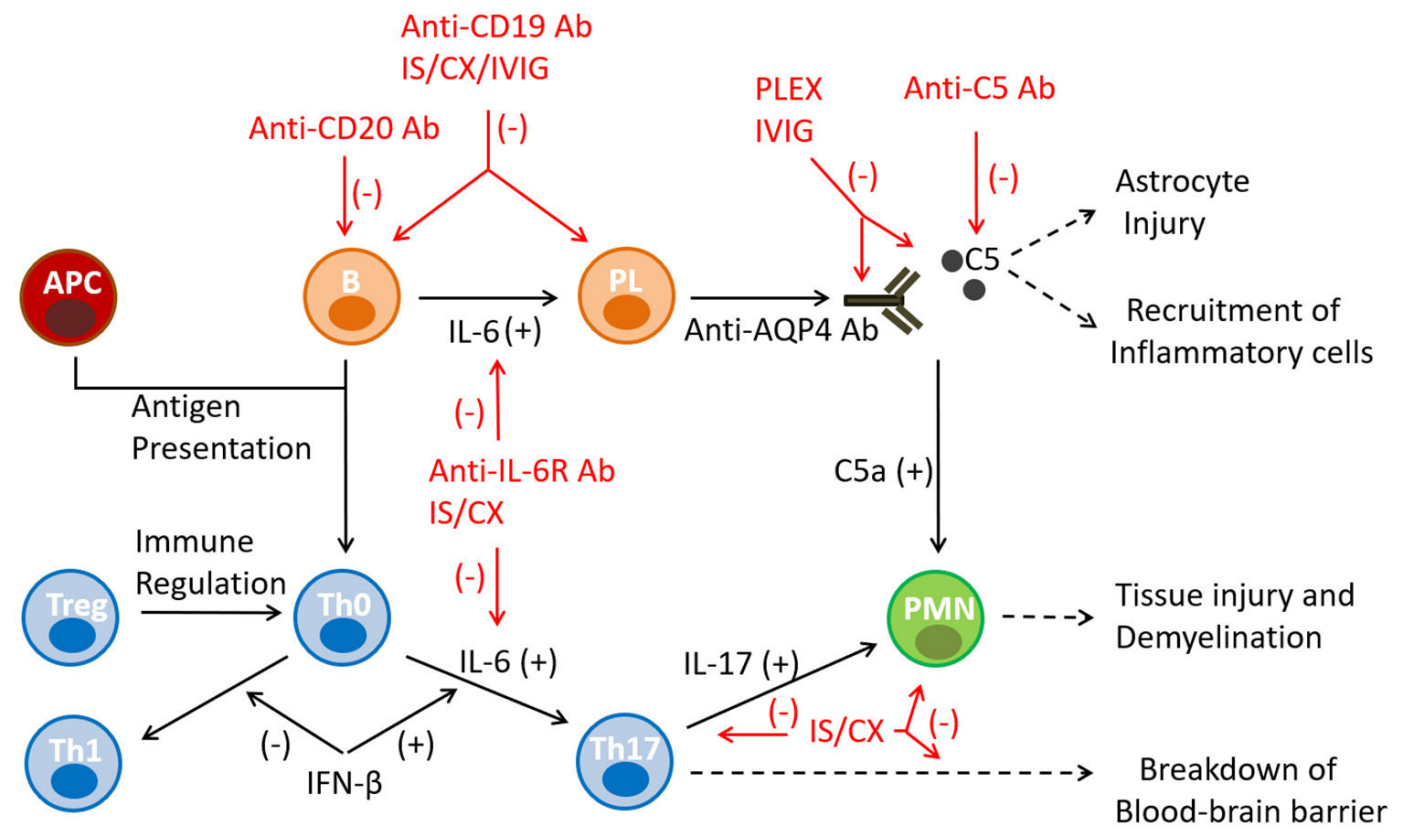
Mechanisms of action of immunotherapies in NMO. The autoimmune reaction in NMO arises in the secondary immune organs as a result of a failure of immune tolerance, pro-inflammatory antigen presentation, and emergence of anti-AQP4 antibody and Th17 cells. The inflammatory reaction evolves in a cascade-like fashion and utilizes several points of augmentation, diversification and integration. Monoclonal antibody therapies target B-lineage cells (anti-CD19, anti-CD20) and the humoral effector molecules/receptors (anti-complement 5, anti-IL-6 receptor) mediating the principal pathway of the disease pathogenesis. Conventional immunosuppressants are non-specific and exert broader cytotoxic effects on the immune cells. APC, antigen presenting cells; Ab, antibody; B, B cell; C5, component 5 of complement; CX, cytotoxic agent; IL, interleukin; IS, immunosuppressant; IVIG, intravenous immunoglobulin; PL, plasma cell; PMN, polymorphonuclear cell; Th0, T helper 0 cell; Th1, T helper 1 cell; Th17, T helper 17 cell; Treg, T regulatory cell.

Immunotherapy of NMO is based on the current understanding of its pathogenesis (Figure 2). The autoimmune reaction arises in the periphery with the emergence of anti-AQP4 antibody and Th17 cells and evolves in a cascade-like fashion. It appears that there are several points of augmentation and diversification of the autoimmune reaction, including complement activation and release of IL-6 and IL-17 (6, 7). These pro-inflammatory and chemotactic factors also contribute to the integration of the T and B cell pathways and the polymorphonuclear cell responses. Inflammation in NMO is an extensive and necrotizing process that can lead to permanent disability with little opportunity for functional recovery. Overall, the neurological disability in NMO represents a cumulative result of the disease relapses and lacks the secondary progressive characteristics that are typically seen in MS (54). Consensus in NMO management favors a timely diagnosis and an early treatment with DMTs, in order to effectively prevent disease relapses, and minimize accumulation of irreversible disability.

In clinical practice NMO can be treated by employing two immunotherapeutic strategies: rescue treatment of an acute disease relapse and prophylactic disease modification (10, 11, 55). Rescue treatment is based on the timely use of corticosteroids and plasmapheresis. Its goal is to rapidly interrupt the inflammatory process to allow functional recovery of the injured tissue. Corticosteroids exert global immunosuppressive and anti-inflammatory effects, whereas plasmapheresis removes antibodies, complement and cytokines from the peripheral blood. The effect of both treatment modalities is rapid, and can be noted within days of their initiation. Alternatively, the purpose of prophylactic disease modification is to prevent future disease relapses and maintain disease remission. DMTs consist of two approaches, monoclonal antibodies and conventional immunosuppressants. Monoclonal antibodies typically target B cell lineage cells (anti-CD19, anti-CD20 antibodies) and the humoral effector molecules (anti-IL-6 receptor, anti-complement 5 antibodies), which medicate the principal pathogenic pathway of the disease (55–57). Conventional immunosuppressants are non-specific and exert broader cytotoxic effects on the immune cells. This category includes chemotherapeutics with antimetabolic (azathioprine, mycophenolate mofetil, methotrexate), alkylating (cyclophosphamide) and topoisomerase II inhibiting (mitoxantrone) properties (55–57). All DMTs are relatively slow acting medications and require chronic administration to induce and maintain their pharmacological effects.

Currently, there are three therapeutic monoclonal antibodies approved by the Food and Drug Administration (FDA) for treatment of anti-AQP4 seropositive NMO (58–60). Eculizumab (Solaris^®^) binds to the component 5 complement protein, and prevents the formation of the C5b-C9 terminal complex and its insertion into the cellular membrane leading to cell lysis. The PREVENT clinical trial demonstrated that eculizumab reduced the relative risk of disease relapse by 94% compared to control; though, there were discrepancies between the investigator-defined and committee-defined quantitations of disease relapses (58, 61). The onset of the effect was rapid and remained present until the completion of the study. Satralizumab (Enspryng^®^) is an IL-6 receptor antagonist and suppresses Th17 differentiation, B cell maturation and antibody production. Its mechanism of action is similar to that of tocilizumab, but it has a longer half-life. The SakuraSky and SAkuraStar clinical trials with the medication determined overall reduction of the risk for disease relapse by 74–78% compared to control (59, 62, 63). Inebilizumab (Uplizna^®^) is a cytolytic antibody that depletes CD19 positive B cells and downregulates the humoral immune response. Its effect on B lineage cells is broader than that of the anti-CD20 (rituximab) therapies and includes antibody-producing plasma cells. The N-momentum trial demonstrated that inebilizumab decreased the relative risk of disease relapse by 77% compared to control (60, 64).

Adverse effects of these medications are predictable and related to their mechanism of action and duration of exposure (58–61). Eculizumab interferes with the processes of bacterial opsonization and phagocytosis, and it is contraindicated in patients with active *N. meningitis* or those who are not vaccinated against the infection. It has a black box warning for life threatening and fatal meningococcal infections (58). Other side effects and complications include respiratory infections, especially with encapsulated bacteria, and infusion reactions. The adverse effects of Satralizumab consist of upper respiratory infection, neutropenia, hypersensitivity, hepatotoxicity and infusion reactions (59). Use of this medication is contraindicated in patients with active hepatitis B infection and untreated latent tuberculosis. Inebilizumab has similar contraindications and it has warnings about common and opportunistic infections, hypogammaglobinemia, neutropenia, life vaccine reversion and fetal risks (60). Decisions regarding the use of a particular DMT are based on pre-treatment risk stratification and implementation of mitigation strategies. However, the latter reflects the current short-term experience with the medications since their long-term side effects are still unknown.

The three FDA-approved monoclonal antibodies are not indicated for treatment of seronegative NMO, though in certain clinical settings they might be of benefit. They are also not indicated for anti-MOG antibody positive NMOSD or MOGAD. Overall the treatment approach to seronegative NMO patients is not standardized and follows the strategies of empirical use of immunotherapy and immunosuppression. This is due to the fact that the pathogenic mechanisms of seronegative NMO are poorly understood and potentially different from our current understanding of the seropositive form of the disease. Additionally, seronegative NMO is likely heterogeneous in nature, creating challenging issues in clinical trial design and efficacy analysis.

Although NMO DMTs target cellular and humoral processes active in the disease pathogenesis, they remain non-specific in nature and only partially effective. DMTs are not antigen-specific or disease-specific, and none of them aim for the eradication of the self-reactive immune cells or result in anti-AQP4 seroreversion. Importantly, their efficacy is based on establishing a state of chronic immunosuppression, where all immune responses are altered, including those protective against infections and cancer. They can also have other off target effects, resulting in a wide range of systemic adverse reactions and treatment complications. The partial clinical benefits of these DMTs and their extinguishable biological effects require life-long administration, while their immunosuppressive characteristics make them unsuitable for risk modification and primary prevention/reversal of the disease. Finally, the substantial financial cost of the newest DMTs particularly that of the monoclonal antibodies, is a frequent limiting factor for their use as a first line therapy or incorporating them in more advanced treatment strategies such as a medication sequencing or a combination therapy.

## Immune Tolerance—A Rationale

Immune tolerance is a state of immune unresponsiveness where an immunogenic molecule cannot elicit an immune response even though the immune system is otherwise normal (12). This is an active process that consists of deletion of self-reactive clones in the thymus and the bone marrow during early development (central tolerance), and downregulation of the self-reactive immune responses in the secondary lymphoid organs throughout life (peripheral immune tolerance) (65–67). Immune tolerance is a critical physiological process that controls “self” from “non-self” discrimination, reduces damage of normal inflammatory processes, protects pregnancy and the fetus, and allows formation of microbiome and bacterial commensalism (67). Autoimmunity is defined as a failure of immune tolerance and uncontrolled activation of self-reactive immune responses (68, 69). Autoimmune diseases are believed to be induced by an environmental trigger, an infectious or a physical agent, in genetically susceptible individuals (69). Genetic predisposition is most associated with HLA alleles, which can either present autoantigens with greater efficiency or compromise the negative selection of self-reactive T and B cell clones (69). Current models of autoimmune diseases also require exposure to cross-immunoreactive antigens, either foreign or altered self proteins, and breakdown of the T- and B cell regulatory mechanisms (68, 69). The primary outcome of the initial autoimmune step is the generation of self-reactive T helper cells, which in turn can subsequently initiate cellular or humoral autoimmune responses (69).

As described above, the immunopathogenesis of NMO, follows our current understanding of autoimmune disease pathogenesis. There are several additional points relevant to NMO that should be considered. In general, B cell immune tolerance is less strict and the processes of B cell receptor editing and affinity maturation provide an escape mechanism from immune regulation (70). Anti-AQP4 IgG is an isotype switched antibody and its production is T cell-dependent, suggesting that the failure of immune tolerance likely also involves T helper cells (10, 15, 71). Association of NMO with connective tissue or paraneoplastic diseases points to possible co-activation of the autoimmune pathways, antigen alteration and epitope spreading (6–8). For instance, Sjögren's syndrome that is co-existent in some patients with NMO, typically targets the salivary glands, which express AQP4 and AQP5 at high levels (72). B cell autoimmunity is much more common in females and characteristically exacerbated by pregnancy (73). In corroboration, NMO is aggravated by pregnancy, an effect attributed to estrogen and its upregulating properties on B cells activating factor (BAFF) and interferon type I, and stimulation of Th2 immune deviation (74, 75). In addition, NMO affects middle-aged individuals suggesting the potential role of more global processes such as immune senescence and age-dependent decline of T regulatory cells (Tregs) (76). Hence, in such settings self-reactive T and B cells controlled by clonal anergy, immunological ignorance or Tregs would be more susceptible to uncontrolled immune activation.

Strategies for restoring immune tolerance in NMO have been previously reviewed (Table 1) (14, 15). The guiding principles aim at elimination of self-reactive T cells by inverse DNA vaccination, autoreactive T cell vaccination, and T cell receptor engineering, or reprogramming the processes of antigen presentation by using tolerogenic dendritic cells or apoptotic antigen-coupled APCs to favor induction of autoantigen-specific T cell anergy and activation of Tregs (14). Other strategies include oral tolerance, stimulation of T and B regulatory (Breg) cell activity, adoptive transfer of autologous Tregs, and administration of anti-idiotypic, or anti-AQP4 blocking antibodies. More recent reports provided direct experimental evidence for the ameliorative effects of adoptive transfer of Treg and antigen-loaded liposomes in animal models of NMO (77, 78). Both studies give support to the significance of immune tolerance in disease regulation and to its potential use as a therapeutic modality. Interest in the immune tolerance approach for treatment of NMO stems from the fact that the autoimmune response is directed against a well-defined antigen and the anti-AQP4 antibody is simultaneously an effector molecule as well as a biomarker marker of the disease, thereby providing a direct measure of treatment efficacy. There are also obvious theoretical and practical challenges with this approach related to its biotechnological feasibility, scarcity of human research data, heterogeneity of NMO, prior exposure to immunotherapy and level of neurological disability. Another important issue is the identification of the disease state or immunological phenotype that is most amendable to immune re-tolerization. Furthermore, it is still unclear what the impact of immune senescence would be when the thymic input becomes limited, and the immune repertoire is comprised mostly of memory cells.

**Table 1:** Strategies for induction of immune tolerance in neuromyelitis optica.

**Method**	**Mechanism of action**
Inverse DNA vaccination	Vaccination with autoantigen-encoding DNA to attenuate activity of autoreactive B and CD8+ T cells.
Anti-autoreactive T cell vaccination	Vaccination with receptor idiotype-restricted CD41/ CD251/FoxP3 Tregs, IL-10-secreting CD41 Tr1cells, and CD81 cytotoxic T cells to modulate and reduce the activity of autoreactive T cell clones.
Dendritic cell vaccination	Administration of immature dendritic cells engineered to maintain a tolerogenic phenotype to inhibit Th1 and Th17 cells and to induce Tregs production of IL-10.
Antigen-coupled apoptotic leukocytes or liposomes	Administration of antigen-apoptotic cell (APC or PMN) or liposome complexes to induce tolerogenic antigen presentation, T cell anergy and upregulation of Tregs.
T cell receptor engineering	Engineering of T cell receptor (TCR) to express a single chain variable fragment from a known antibody to prevent off-target major histocompatibility complex (MHC) restriction.
Regulatory T cell induction	Administration of autologous polyclonal CD4+CD25+Foxp3+ regulatory T cells to modulate immune responses to autoantigens.
Regulatory B cell induction	Adoptive transfer of TGF-β-producing B cells (Bregs) to attenuate disease related autoimmunity by targeting pathogenic cells and secretion of IL-10.
Oral/mucosal tolerization	Oral administration of autoantigen to stimulate gut-associated T regulatory cells.
Adoptive transfer	Adaptive transfer of AQP4-specific T and B cells to recipients for the purpose to modulate pathogenic effector cells *via* IL-10 and TGF-β.
Anti-idiotypic networks	Targeting of antigen-binding Fab domains of anti-AQP4 antibody by recombinant anti-idiotypic antibodies.
Passive tolerization	Therapeutic use of aquaporumab, a recombinant monoclonal antibody that functions as a competitive inhibitor to anti-AQP4 antibodies because of its high affinity for AQP4 and lack of cytotoxic properties.
Hematopoietic stem cell transplantation	Immune ablative therapy with hematopoietic stem cell rescue aiming to destroy the autoimmune clones and to induce immune reset and long- term immune tolerance.
TIMP	Intravenous administration of tolerogenic immune-modifying PLG nanoparticles encapsulating autoantigen to induce specific T cell anergy and upregulation of iTregs and Tr1 cells.

Safety and tolerability of peptide-loaded tolerogenic dendritic cells was recently evaluated in a phase Ib clinical trial (16). Eight patients with multiple sclerosis and four with NMO received between one and three intravenous treatments with dendritic cells loaded with myelin or APQ4 peptides, respectively, over a period of 12 weeks. Tolerogenic dendritic cells were generated from each individual patient in the presence of several cytokines, dexamethasone and the respective peptides. The intended tolerogenic phenotype of the cells was ascertained by their immature state of development and increased production of IL-10. The end of study evaluation performed at 24 weeks demonstrated that the treatment was well-tolerated and not associated with significant adverse effects, or changes in vital signs, blood cell count and chemistry. Patients remained clinically stable without new disease relapses, worsening of neurological disability, or quality of life. The immunological response to the treatment, though not an outcome measure, was also examined. The most important finding was that peripheral blood mononuclear cells produced significantly higher levels of IL-10 compared to baseline in all patients. Trends toward increased Tr1 (T regulatory type 1) cells and decreased T cell proliferation in response to AQP4 were observed as well. The overall conclusion of the study was that treatment with peptide-loaded tolerogenic dendritic cells appeared to be a safe and feasible approach for induction of immune tolerance.

Studies using autologous hematopoietic stem cell transplantation (HSCT) brought an important new perspective on immune tolerance in NMO. An open label prospective study involving 11 AQP4 positive patients with NMOSD demonstrated several significant findings (79). HSCT with a conditioning regimen consisting of cyclophosphamide, anti-thymoglobulin, and rituximab, was sufficient to induce long-term disease remission and to improve neurological disability of patients. Disease remission occurred in 9 out of the 11 patients and it was associated with conversion to an anti-AQP4 seronegative status. Conversely, patients who remained positive for the autoantibody relapsed during the study. Three patients who entered disease remission, became seropositive again, though at low levels, one of them transiently and the other two permanently. Importantly, the re-emergent anti-AQP4 antibody in the last two patients appeared to be atypical as it lacked complement fixation and cell killing properties. Theoretically, HSCT might have induced an immune reset consisting of deletion of the T and B self-reactive clones and restoration of AQP4-specific immune tolerance that prevented their re-appearance. It is also reasonable to consider that the Treg control on affinity maturation and clonal expansion of B cells has been restored. The observed treatment efficacy of the study was superior compared to that reported by others whose conditioning regimen did not contain rituximab (80). Still, the use of rituximab is not sufficient to explain the elimination of the anti-AQP4 antibody since the medication alone does not induce seronegativity even when it is titrated to the point of elimination of B memory (CD27+) cells (81).

## Tolerogenic Immune-Modifying Nanoparticles

Tolerogenic immune-modifying nanoparticles (TIMP) are a novel therapeutic strategy of inducing T cell tolerance using the intravenous administration of biodegradable carboxylated nanoparticles coupled with or encapsulating protein or peptide antigen(s) (17). The 500 nm particles are comprised of poly(lactide-*co*-glycolide) (PLG) consisting of lactic and glycolic acids, both natural metabolites. Thus, the break-down products of the PLG are non-toxic and eliminated through metabolic pathways. PLG is commonly used in the production of biodegradable sutures. PLG nanoparticles are FDA-approved for controlled delivery of drugs, proteins, DNA vaccines and other molecules of commercial and research interest (17). Encapsulating or conjugating the (auto)antigen(s) with PLG nanoparticles is a relatively uncomplicated process making the TIMP strategy versatile and advantageous in terms of disease targeting, pharmacological design, and production scale.

**Figure 3:**
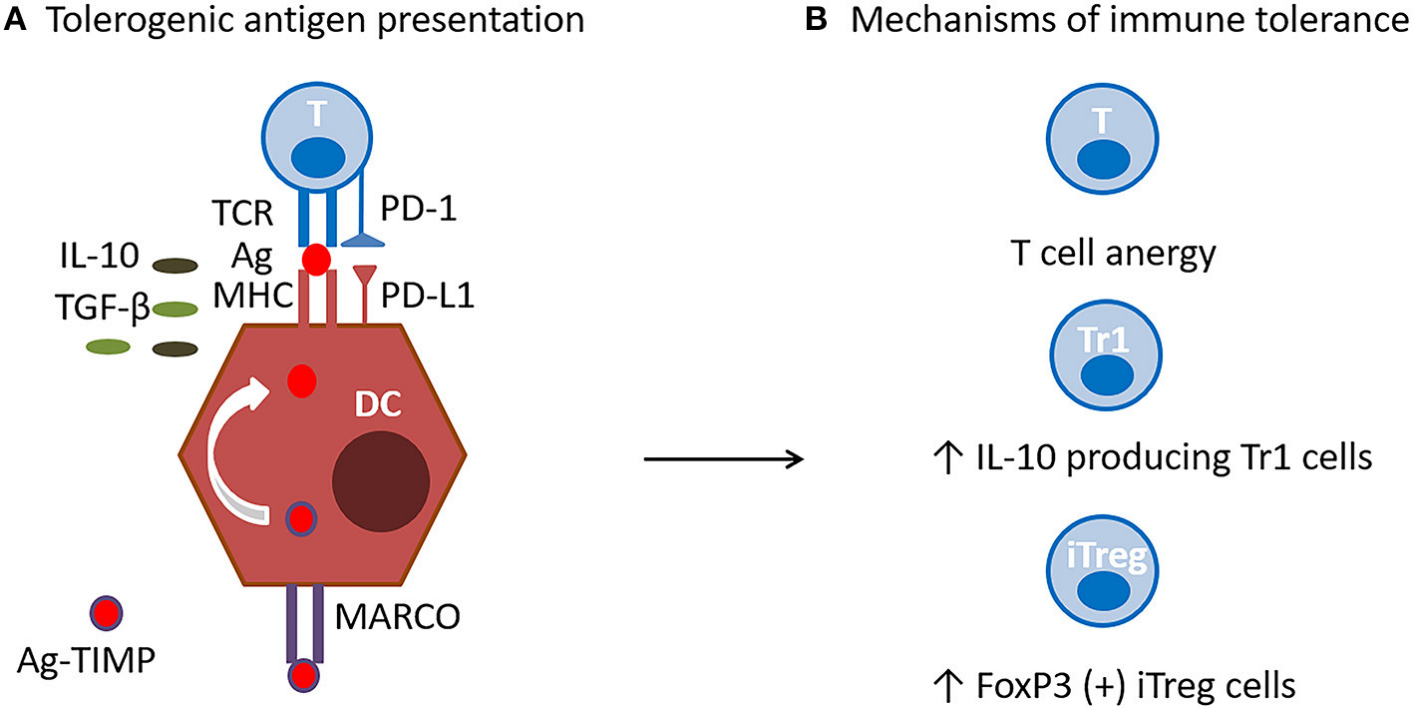
TIMP Mechanism of action. **(A)** Tolerogenic antigen presentation. Antigen encapsulating PLG nanoparticles (Ag-TIMP), when administered intravenously, are taken up by MARCO+ antigen presenting cells (APC) localized in the liver and splenic marginal zone. Tolerogenic antigen presentation requires interactions between antigen (Ag), T cell receptor (TCR) and major histocompatibility (MHC) class II molecule in the presence of anti-inflammatory molecules. Engulfment of the nanoparticles induces the APC expression of PD-L1, and secretion of TGF-β and IL-10, which have immunomodulatory and anti-inflammatory functions. **(B)** Mechanisms of immune tolerance. Effective immune tolerance is mediated by induction of antigen-specific T cell anergy, and activation of iTregs and Tr1 cells.

TIMP target antigen presenting cells (APC) localized in the liver and marginal zone of the spleen, and are engulfed *via* an immunological system designed to dispose of apoptotic cell debris and self-antigens, while preventing immune activation (17). The tolerogenic effects of nanoparticles depend on their intravenous administration, APC uptake, and induction of expression of tolerogenic molecules. APC uptake is primarily mediated by the MARCO (macrophage receptor with collagenous structure) scavenger receptor, whose affinity for polyanionic macromolecules represent an ideal target for the negatively charged PLG nanoparticles. Receptor interaction and the engulfment of the nanoparticles induces the cellular expression of PD-L1 (program death-ligand 1) and secretion of TGF-β (transforming growth factor beta), and IL-10. Effective immune tolerance is mediated by tolerogenic antigen presentation, induction of antigen-specific T cell anergy, activation of iTreg (FoxP3+ T regulatory cells), and Tr1 (T regulatory cells expressing IL-10) (Figure 3). It is still unclear if the TIMP would have the capacity to induce or modulate Bregs or whether their effects are restricted to regulation of T cell function.

The TIMP strategy was developed after multiple years of research testing different methods of immune tolerance induction and polymer chemistry (17, 82, 83). Originally, tolerance was induced by chemically coupling the autoantigens to syngeneic donor apoptotic leukocytes using 1-ethyl-3-(3-dimethylaminopropyl) carbodiimide (ECDI), and the antigen-apoptotic cell conjugates were utilized to drive APC toward tolerogenic antigen presentation (82, 83). This immune tolerance strategy was tested initially in experimental autoimmune encephalomyelitis (EAE) and subsequently in a Phase 1 clinical trial in MS (83, 84). In the clinical trial, a single infusion of autologous peripheral blood leukocytes conjugated with multiple myelin peptides (MOG_1−20_, MOG_35−55_, MBP_13−32_, MBP_83−99_, MBP_111−129_, MBP_146−170_, and PLP_139−154_) was administered to 9 patients with MS (84). The study demonstrated clinical feasibility, good tolerability and favorable safety. Also, none of the patients developed a disease relapse or symptoms of clinical worsening. Importantly, it also indicated a dose-dependent reduction of the antigen-specific T cell responses. The positive results of the study eventually led to the development of autoantigen-encapsulated PLG nanoparticles as a way to standardize the treatment process and to allow for large scale clinical studies, and implementation in clinical practice.

In pre-clinical studies, PLG nanoparticles with encapsulated myelin antigens were found to be effective in treating and preventing EAE (85, 86). Disease suppression correlated with significant reduction of encephalitogenic Th1 (IFN-γ) and Th17 (IL-17a, GM-CSF) cells as well as of inflammatory monocytes/macrophages (85, 86). These experiments further revealed that the beneficial effect of nanoparticles on EAE was in fact due to induction of antigen-specific immune tolerance and not to immunosuppression. The experiments were performed with negatively charged PLG nanoparticles with average size of 500–1,000 nm, which when administered intravenously, would rapidly reach the MARCO+ APCs in the marginal zone of the secondary immune organs (85, 86). Importantly, the physico-chemical properties of the nanoparticle (size and negative charge) allowed for efficient engagement of MARCO receptor and activation of the innate scavenger pathway under its control. In essence, nanoparticles functioned as a surrogate for apoptotic cells and cell debris that are capable of programming the tolerogenic properties of APCs.

A recent phase I/Ila randomized, double blind, placebo-controlled clinical trial in celiac disease (sponsored by Cour Pharmaceuticals and Takeda Pharmaceuticals) using the TIMP strategy provided proof-of-concept that antigen-specific immune tolerance could be safely induced in patients with a pre-existing autoimmune disease without causing systemic immunosuppression (87). Celiac disease is an autoimmune disorder caused by an inflammatory damage to the intestinal walls as a result of intolerance to gluten, and more specifically, to one of its peptide components gliadin (88). A Phase I (dose finding) study determined that 8 mg/kg of PLG nanoparticles encapsulating purified gliadin (TIMP-GLIA, TAK-101) administered intravenously over 30 min was safe and well-tolerated. Pharmacokinetic analysis revealed a mean terminal half-life of gliadin-nanoparticle between 2 and 4 h without a residual body/tissue accumulation. The adverse effects were limited to transient infusion reactions and a single case of self-remitting colitis that did require medical treatment.

The Phase IIa efficacy study involved 34 patients with well-controlled celiac disease, who were divided in two cohorts in 1:1 ratio (87). Also, by design, the study required a baseline pre-treatment disease assessment at day-45 that included clinical examination, bowel biopsy and laboratory testing. Treatment itself involved two infusions with either gliadin- encapsulated nanoparticles (TAK-101) or placebo at days 1 and 8. The treatment was followed, a week later, by a 14-day oral gluten challenge (13 g/day for 3 days and 6 g/days for 11 days) and pre-scheduled clinical and laboratory assessments and an end-of-study repeat bowel biopsy. Expectedly, gluten challenge caused a rapid increase of IFN-γ, gliadin-specific T cells responses of peripheral blood T cells, mucosal inflammation and villous atrophy of the colon in the placebo-treated cohort. In contrast, gliadin-specific T cells responses the TAK-101- treated cohort were reduced nearly 90%, while responses to anti-CD3 were equivalent to those seen in the placebo controls. The impact on inflammatory pathology, though not clinically significant, demonstrated a strong trend toward small bowel protection. In terms of adverse effects, the gliadin-nanoparticle treatment was well-tolerated and within the spectrum of infusion reactions and gluten intolerance, the majority of them mild to moderate in severity. One patient discontinued the study for non-compliance with the treatment protocol. There were no severe adverse effects or death during the study. Overall, the results of the clinical trial demonstrated that TIMP is a feasible strategy for induction of immune tolerance and that it appears to be safe and not associated with systemic immunosuppression. In addition, the TIMP has the capacity to induce its tolerogenic effects fairly rapidly, i.e., within a week after only two TAK-101 infusions. Our laboratory is currently testing the ability of PLG nanoparticles encapsulating either the immunodominant AQP4 T cell peptide epitope or full-length recombinant AQP4 to both prevent and treat ongoing clinical disease in experimental animal models of NMO (89, 90). Thus, clinical testing of immune tolerance to AQP4 and MOG in NMO is anticipated to begin in the near future.

## Conclusion

Immunotherapy of NMO continues to evolve and to adapt to the new clinical and research advancements related to enhanced understanding of the disease pathogenesis. The goals and expectations of immunotherapies have shifted from accepting partial efficacy to achieving a state of long-term remission. Not surprisingly, the many successes in the field of NMO raise the question of disease cure rather than non-specific suppression of the pathogenic effector responses. Certainly, a long-term disease reversal necessitates the re-establishment of autoantigen-specific immune tolerance. There is sufficient experimental and clinical evidence as well as biotechnological feasibility in support of such a strategy, particularly employing the TIMP strategy. Development and implementation of immune tolerance-based therapies in NMO are very likely to improve the treatment outcomes of the disease, and limit the clinical challenges associated with tolerability, chronic immunosuppression and cost of treatment. Immune tolerance-based therapies will also enrich the immunotherapeutic strategies in general and allow for combination therapies or medication sequencing, as well as eventually allowing pre-disease prophylaxis in genetically susceptible patients. Successful immune tolerance therapy would have far reaching consequences and clinical implications within the entire spectrum of autoimmune diseases.

## Author Contributions

EL and RB contributed the primary drafting and conception of the work. GA and GP-G contributed in additional drafting and editing of the review. SM provided critical revisions to the article. RB and SM gave final approval for submission. All authors contributed to the article and approved the submitted version.

## Funding

This work was supported by NIH Grants R01 NS099334 and R21 AI142059, and by the Johnnie Walker's MS Foundation, the David and Amy Fulton Foundation, and the Cramer Family Foundation.

## Conflict of Interest

SM reports being a co-founder, a member of the Scientific Advisory Board, and a grantee of and holds stock options in COUR Pharmaceuticals Development Co.; being a paid consultant for COUR Pharmaceuticals Development Co. and Takeda Pharmaceuticals International Co; being a paid consultant and member of the Scientific Advisory Board of NextCure Inc.; being a paid consultant for Kite Pharmaceuticals; and being a paid consultant for and member of the Scientific Advisory Board of Myeloid Therapeutics. The remaining authors declare that the research was conducted in the absence of any commercial or financial relationships that could be construed as a potential conflict of interest.

## Publisher's Note

All claims expressed in this article are solely those of the authors and do not necessarily represent those of their affiliated organizations, or those of the publisher, the editors and the reviewers. Any product that may be evaluated in this article, or claim that may be made by its manufacturer, is not guaranteed or endorsed by the publisher.
